# Racial differences in longitudinal toxicities of anticancer agents in early phase cancer clinical trials

**DOI:** 10.1002/cam4.6370

**Published:** 2023-07-31

**Authors:** Junki Mizusawa, Hioryuki Sato, Larry V. Rubinstein, Takeo Fujiwara, Kan Yonemori, Akihiro Hirakawa

**Affiliations:** ^1^ Biostatistics Section, Clinical Research Support Office, National Cancer Center Hospital/Biostatistics Division, Center for Research Administration & Support National Cancer Center Tokyo Japan; ^2^ Department of Global Health Promotion Tokyo Medical and Dental University Tokyo Japan; ^3^ Department of Clinical Biostatistics, Graduate School of Medical and Dental Sciences Tokyo Medical and Dental University Tokyo Japan; ^4^ Biometric Research Program, Division of Cancer Treatment and Diagnosis, National Cancer Institute National Institute of Health Rockville Maryland USA; ^5^ Department of Breast and Medical Oncology National Cancer Center Hospital Tokyo Japan

**Keywords:** cancer chemotherapy, longitudinal toxicity, racial differences

## Abstract

**Background:**

Racial differences have been reported in toxicity outcomes for anticancer drug treatments. However, these observations were often from studies with small sample sizes, and many only reported the maximum grade of toxicity and no longitudinal information. This current analysis aims to investigate racial differences in longitudinal toxicities using a large‐scale clinical trials database.

**Methods:**

Early‐phase clinical trials sponsored by the Cancer Therapy Evaluation Program at the National Cancer Institute, USA, that evaluated cytotoxic drugs and molecularly targeted agents between March 2000 and December 2012 were studied. Race was categorized as White, Black or African‐American, and Asian. Each toxicity's grade prevalence, mean grade at each cycle, and time to develop grade 2 or higher toxicity was evaluated.

**Results:**

In total, 25,442 patients from 697 trials were included in this study. The number of patients categorized as White, Black, and Asian designations was 22,756 (89%), 1874 (7%), and 812 (3%), respectively. Notable findings include the rate of any grade of diarrhea in Black people was 26% and 21% lower than that of White and Asian people. The median time to the first grade 2 or higher event was 6 cycles in White people, 8 in Black people, and 6 in Asian people. The rate of any grade hyperglycemia was significantly higher in Asian people.

**Conclusions:**

Although we identified several racial differences in longitudinal toxicities, most were of generally lower grade. Further study is needed to clarify the cause of racial differences in treatment‐associated toxicities.

## Graphical abstract

This study analyzed a large clinical trial database to investigate racial differences in longitudinal toxicities of cancer treatments. Notable findings include lower rates of diarrhea in Blacks and longer time to first grade 2 or higher event in Blacks, whereas the rate of hyperglycemia was higher in Asians. However, most differences were generally of lower grade and further study is needed to clarify the cause of these differences.

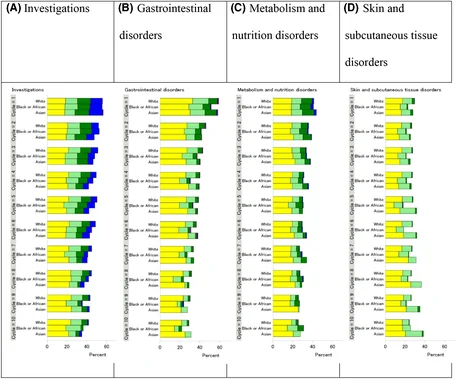

## 1 INTRODUCTION

Cancer is one of the most important causes of mortality globally.^1^ In the United States, the overall cancer incidence rate per 100,000 population in 2020 was 401.1 for all races, 407.0 in White people, 393.0 in Black people, and 270.0 in Non‐Hispanic Asian/Pacific Island people.^2^ It has been recognized that different races may have different treatment and adverse events outcomes for anticancer drug treatments.^3^, ^4^, ^5^, ^6^, ^7^, ^8^, ^9^, ^10^, ^11^, ^12^ However, the results have not been consistent, partly due to barriers in access to culturally competent care for underrepresented patients^13^ and to adverse social determinants of health such as geographic and cultural factors, access to healthcare, and financial barriers.^14^

For example, Irinotecan, a drug used to treat colorectal cancer, causes diarrhea as one of its major adverse events, which was observed to be more common in Asian people than in White people or Black people.^15^, ^16^ Patients homozygous for the UGT1A1*6 and *28 alleles were highly sensitive to Irinotecan, often displaying severe myelosuppression. The frequency of the *6 allele is higher in Asian people than in White people and Black people.^15^, ^16^ McCollum et al. reported that African‐American people experienced statistically significantly lower rates of diarrhea (51% vs. 75%), nausea (47% vs. 61%), vomiting (24% vs. 30%), stomatitis (42% vs. 59%), and overall toxicity (44% vs. 51%) compared with White people for resected colon cancer who received adjuvant 5‐fluorouracil‐based chemotherapy in a study involving 3380 patients.^9^ On the other hand, Polite et al. found in 1816 patients that arterial thromboembolic events were no different between African‐American people and White people with metastatic colorectal cancers receiving first‐line bevacizumab therapy.^11^ Overall, these effects were often shown in small‐scale studies within a single disease‐specific trial with a particular drug.

Common limitations of previous investigations were that these studies typically only reported the maximum grade of adverse effects (AE) during treatment in each subject with no longitudinal toxicity information. In addition, most studies examined racial differences between two races, for example, Black and White people, and there were no large‐scale cohort studies investigating racial differences among Black, White, and Asian people. Black and Asian people, in particular, often have small sample sizes, so studies using large‐cohort sizes are needed to examine racial differences in toxicity. Some adverse events may be caused by genetic variations, but genetic variation and genome‐wide association studies have often focused on patients of European descent (predominantly White).^14^ Insufficient research has been conducted on non‐White populations.^14^ It is of utmost importance to evaluate the frequency, timing, and severity of adverse event occurrence among different racial groups as it may necessitate dose adjustments. Therefore, assessing these factors on a subgroup basis is crucial.

Therefore, we aimed to investigate the racial differences among Black, White, and Asian peoples in longitudinal toxicities. Specifically, we examined not only the worst grade of toxicity experienced during treatment but also the worst grade within each treatment cycle by analyzing the average grade or distribution of grades within each treatment cycle. These data were collected from the large database for early phase clinical trials sponsored by the Cancer Therapy Evaluation Program (CTEP) of the National Cancer Institute (NCI), USA.

## 2 METHODS

### 2.1 Study design and patient selection

**FIGURE 1:**
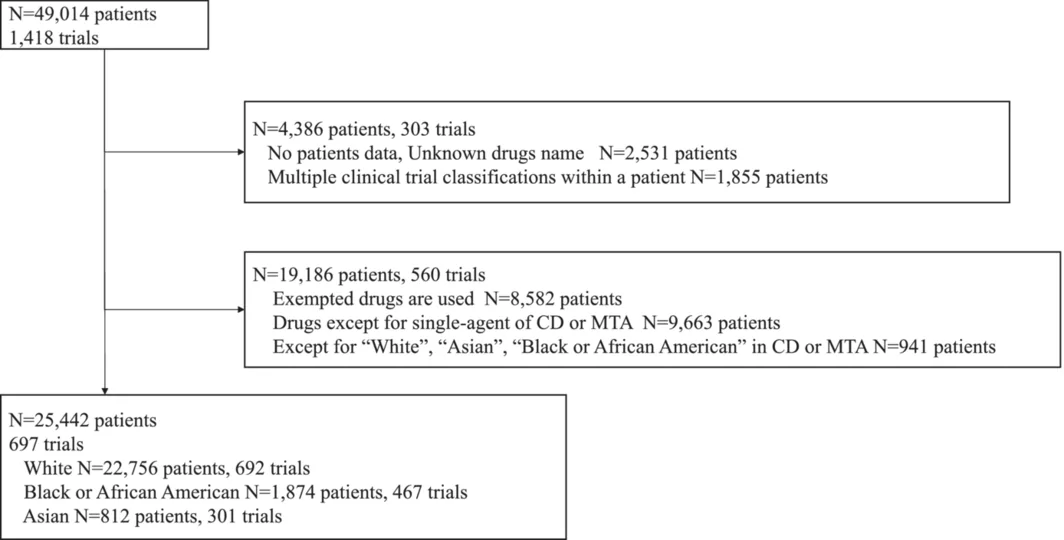
Patients flow diagram for the study. Among the 49,014 patients from 1418 trials, 4386 patients were excluded because of missing data on drug names and/or multiple classifications of trials. In addition, 19,186 patients were excluded due to the use of combination therapies or therapies other than molecularly targeted agents and cytotoxic therapy and due to racial designations other than White, Asian, and Black or African‐American. The remaining 25,442 patients from 697 trials that fulfilled our study criteria were analyzed.

We investigated the patients enrolled in CTEP‐sponsored early phase (phase I, phase I/II, phase II) clinical trials for oncology initiated between March 2000 and December 2012 to evaluate the efficacy and safety of the investigational drugs. As shown in Figure 1, among the 49,014 patients from 1418 trials, 4386 patients were excluded because of missing data on drug names or multiple classifications of trials. Furthermore, a total of 19,186 patients were excluded from the analysis. These exclusions were made based on the following criteria: (1) the use of combination therapies, as their toxicity evaluation is more complex compared with monotherapies; (2) the use of therapies other than molecularly targeted agents and cytotoxic therapy, as they had limited sample size and a smaller number of available options; and (3) race designations other than White, Asian, and Black. The remaining 25,442 patients from 697 trials that fulfilled our study criteria were analyzed. Categorization of investigational drugs into chemotherapy or molecularly targeted agents was done by medical experts as described previously.^17^, ^18^, ^19^ An analyzed drug list containing 68 drugs, 21 chemotherapeutic drugs (CD) and 47 molecularly targeted agents (MTA), is shown in Table S1.

### 2.2 Data collection

The data from the early phase trials sponsored by NCI/CTEP were extracted prospectively from the Clinical Trials Monitoring System database managed by Theradex Systems. Data collected for each patient included age, race, sex, performance status (PS), trial start and end dates, administered drugs, grading of any toxicities including the protocol‐defined dose‐limiting toxicities (DLTs) occurring in each cycle, and date of occurrence. Data on grades 1–5 toxicities for all cycles of every trial were collected according to the National Cancer Institute/Common Terminology Criteria of Adverse Events (NCI/CTCAE) (version 2.0, 3.0, or 4.0).^20^, ^21^, ^22^ The Medical Dictionary for Regulatory Activities (MedDRA) version 20.1 was used in order to provide a System Organ Class (SOC) and Preferred Term (PT) for each event, which was graded according to the NCI‐CTCAE. A treatment cycle, defined in the corresponding protocol of the trial, was used as the time unit irrespective of the duration of treatment in terms of the number of days.

Toxicity was classified based on SOC for each event into the following categories: (i) investigations, (ii) gastrointestinal disorders; (iii) metabolism and nutrition disorders; (iv) general disorders and administration site conditions; (v) skin and subcutaneous tissue disorders; (vi) nervous system disorders; (vii) blood and lymphatic system disorders; (viii) respiratory, thoracic, and mediastinal disorders; (ix) musculoskeletal and connective tissue disorders; (x) infections and infestations; (xi) vascular disorders; (xii) psychiatric disorders; (xiii) renal and urinary disorders; and (xiv) cardiac disorders.

### 2.3 Statistical analysis

Race was categorized as either White, Asian, or Black. Continuous variables were summarized with median and range and compared using the Kruskal–Wallis test. Categorical variables were compared using the Chi‐squared test.

Mortality rate, the occurrence of CTCAE grade of one or more toxicities during 10 treatment cycles, and mean CTCAE grade of SOC‐based toxicities during 10 cycles were calculated. Regarding the mean CTCAE grade, adjusted mean grades over all cycles for each toxicity were estimated by a mixed model for repeated measures (MMRM). The maximum grade toxicity was only counted once in each cycle per patient.

In the MMRM, the CTCAE grade over a cycle was regarded as a continuous dependent variable with missing data due to protocol‐off events such as treatment interruption and death. The model included age (continuous), sex (male vs. female), PS (0–1 vs. ≥2), trial phase (1 vs. 1/2 vs. 2), race, cycle, and interaction of race and cycle as explanatory variables. Time to the grade 2 or higher event of interest was defined as the time from the start date of treatment to the cycle that the first occurrence of grade 2 or higher event and was censored on the last cycle within 10 cycles the patient was alive without any event of interest was analyzed using the Kaplan–Meier method. All statistical analyses were conducted using SAS version 9.4.

## 3 RESULTS

### 3.1 Patient characteristics

The number of patients with White, Black, or Asian designations were 22,756 (from 692 trials), 1874 (from 467 trials), and 812 (from 301 trials), respectively.

The baseline characteristics of these patients across races are shown in Table 1. The median age of White people (59 years) was 5 years older than that of Black people (54 years) and 4 years older than that of Asian people (55 years). Black people were about 5% less likely than other races to receive molecularly targeted agents (MTAs). There were no meaningful differences in baseline characteristics regarding PS or sex.

**TABLE 1:** Patient characteristics.

	White (*N* = 22,756, 89.4%)	Black or African (*N* = 1874, 7.4%)	Asian (*N* = 812, 3.2%)	All (*N* = 25,442)
PS				
0	9744 (44%)	765 (42%)	344 (43%)	10,853 (43%)
1	10,870 (49%)	917 (50%)	385 (48%)	12,172 (49%)
2	1674 (7%)	154 (8%)	66 (8%)	1894 (8%)
3	33 (0%)	1 (0%)	3 (0%)	37 (0%)
4	3 (0%)	0 (0%)	1 (0%)	4 (0%)
Age				
*n*	22,756	1874	812	25,442
Median	59	54	55	59
Range	0–94	1–89	2–87	0–94
Age				
≤65	15,116 (66%)	1464 (78%)	624 (77%)	17,204 (68%)
66–70	3151 (14%)	203 (11%)	87 (11%)	3441 (14%)
71–75	2344 (10%)	121 (6%)	50 (6%)	2515 (10%)
76–80	1450 (6%)	54 (3%)	39 (5%)	1543 (6%)
80<	695 (3%)	32 (2%)	12 (1%)	739 (3%)
Sex				
Male	12,136 (53%)	898 (48%)	437 (54%)	13,471 (53%)
Female	10,616 (47%)	976 (52%)	374 (46%)	11,966 (47%)
CD or MTA				
CD	3518 (15%)	371 (20%)	126 (16%)	4015 (16%)
MTA	19,238 (85%)	1503 (80%)	686 (84%)	21,427 (84%)

Abbreviations: CD, cytotoxic drug; MTA, molecularly targeted agent.

### 3.2 Mortality rate and toxicity in the first 10 cycles of treatment

Table 2 shows the mortality rates and toxicity in the first 10 treatment cycles across races. Deaths due to adverse events were often attributed to general disorders and administration site conditions, regardless of race, with rates ranging from 2.2% to 2.8%. There were no major differences among races for these parameters. Other causes of death due to adverse events were gastrointestinal disorders, respiratory, thoracic, and mediastinal disorders, infections and infestations, and nervous system disorders, each of which with rates less than 1%, and these did not differ significantly among races. The proportion of dose reduction during the 10 cycles was 46%–48%, and there were no significant differences among races.

**TABLE 2:** Treatment‐related death rate by race.

	White (*N* = 22,756)		Black or African (*N* = 1874)		Asian (*N* = 812)	
	*N*	%	*N*	%	*N*	%
General disorders and administration site conditions	503	2.2	52	2.8	21	2.6
Gastrointestinal disorders	132	0.6	7	0.4	5	0.6
Respiratory, thoracic, and mediastinal disorders	116	0.5	13	0.7	4	0.5
Infections and infestations	111	0.5	10	0.5	2	0.2
Nervous system disorders	39	0.2	3	0.2	5	0.6
Cardiac disorders	31	0.1	4	0.2	0	0.0
Vascular disorders	21	0.1	4	0.2	1	0.1
Blood and lymphatic system disorders	19	0.1	3	0.2	0	0.0
Renal and urinary disorders	11	0.0	1	0.1	1	0.1
Investigations	3	0.0	2	0.1	2	0.2
Metabolism and nutrition disorders	5	0.0	1	0.1	0	0.0
Psychiatric disorders	2	0.0	0	0.0	0	0.0
Skin and subcutaneous tissue disorders	0	0.0	0	0.0	0	0.0
Musculoskeletal and connective tissue disorders	0	0.0	0	0.0	0	0.0

### 3.3 Occurrence of CTCAE grade one or more toxicities during the first 10 treatment cycles

**FIGURE 2:**
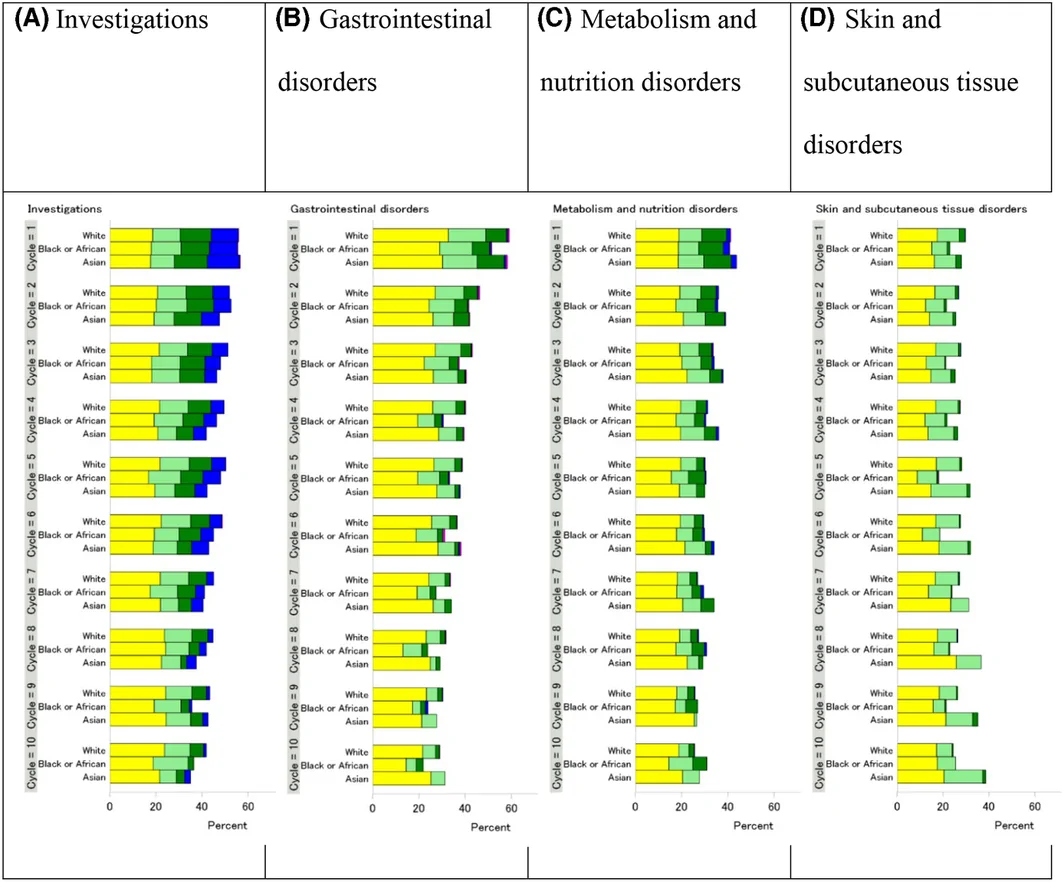
The grade‐by‐prevalence at each cycle. The grade‐by prevalence of each SOC toxicity at each cycle. The maximum grade toxicity was only counted once at each cycle per patient for each toxicity. The colors represent as follows: Yellow, grade 1; lime green, grade 2; green, grade 3; blue, grade 4, pink, grade 5. SOC, System Organ Class.

The occurrence of “CTCAE any grade” toxicities observed in more than 10% of patients during the first 10 treatment cycles is shown in Table 3. As for SOC of investigations, the decreased neutrophil count rate in Black people was 9.2% higher than that of Asian people, whereas other hematological toxicities, including decreased platelet count, decreased lymphocyte count, and decreased white blood cell count rates in Black people were lower than that of White and Asian people. The rates of grade 3 or 4 SOC of investigations gradually decreased from cycle 1 to cycle 10 regardless of race, as shown in Figure 2A. There was no remarkable difference among races, although there was a trend toward a slightly lower frequency of adverse events in Asian people.

**TABLE 3:** Toxicities based on the classifications of System Organ Class (SOC) and Preferred Term (PT) (≥10%).

SOC	PT	White (*N* = 22,756)	Black or African‐American (*N* = 1874)	Asian (*N* = 812)
		%	%	%
Investigations	Neutrophil count decreased	54.7	59.0	49.8
Investigations	Platelet count decreased	30.2	18.9	23.3
Investigations	Lymphocyte count decreased	25.1	17.6	20.4
Investigations	White blood cell count decreased	21.1	19.3	24.8
Investigations	Alanine aminotransferase increased	20.2	19.1	27.6
Investigations	Blood alkaline phosphatase increased	14.1	11.5	11.7
Investigations	Weight decreased	12.0	10.7	7.5
Gastrointestinal disorders	Diarrhea	68.6	42.9	64.2
Gastrointestinal disorders	Nausea	37.8	34.7	33.3
Gastrointestinal disorders	Constipation	22.3	23.5	17.0
Gastrointestinal disorders	Abdominal pain	16.5	18.6	16.4
Gastrointestinal disorders	Stomatitis	14.3	8.8	17.6
Metabolism and nutrition disorders	Decreased appetite	40.0	34.8	47.2
Metabolism and nutrition disorders	Hyperglycemia	34.9	37.3	55.0
Metabolism and nutrition disorders	Hypoalbuminemia	13.0	13.9	11.1
Skin and subcutaneous tissue disorders	Alopecia	39.8	29.5	29.4
Skin and subcutaneous tissue disorders	Rash maculopapular	35.8	19.4	24.1
Skin and subcutaneous tissue disorders	Dry skin	11.4	8.3	18.5
Nervous system disorders	Peripheral sensory neuropathy	34.8	30.6	21.7
Nervous system disorders	Headache	19.6	17.1	14.4
Nervous system disorders	Dysgeusia	13.7	15.2	17.1
Respiratory, thoracic, and mediastinal disorders	Dyspnea	20.2	16.1	10.5
Respiratory, thoracic, and mediastinal disorders	Cough	11.4	10.8	12.7
Musculoskeletal and connective tissue disorders	Arthralgia	17.4	10.5	11.0
Musculoskeletal and connective tissue disorders	Myalgia	12.2	8.9	10.5
Vascular disorders	Hypertension	19.8	21.6	30.0
Infections and infestations	Infections and infestations	15.0	12.5	12.1

*Note*: The percentage indicates the prevalence of patients who developed toxicity at least once during treatment.

In terms of gastrointestinal disorders, the rate of any grade of diarrhea in Black people was 26% and 21% lower than that of White and Asian people, respectively. In all cycles, Black people tended to have lower rates of toxicities than White and Asian people. However, the severity of those who developed gastrointestinal disorders was higher for Black people and typically occurred later in treatment (Figure 2B).

In SOC of metabolism and nutrition disorders, the rate of experiencing decreased appetite in Asian people was 47.2% (95% confidence interval; 43.7%–50.7%), followed by White people (40.0%, [95% confidence interval; 39.4%–40.6%]) and Black people (34.8%, [95% confidence interval; 32.7%–37.0%]). Also, the rate of experiencing hyperglycemia in Asian people (55.0%, [95% confidence interval; 51.6%–58.5%]) was more frequent than in White people (34.9%, [95% confidence interval; 34.3%–35.5%]) and Black people (37.3%, [95% confidence interval; 35.1%–39.5%]). Asian people tended to have a higher frequency of SOC of metabolism and nutrition disorders compared with White and Black people through the seventh cycle. However, Black people experienced higher grades of CTCAE than others at the eighth cycle or later, as shown in Figure 2C. Frequency of skin and subcutaneous tissue disorders of SOC including alopecia, maculopapular rash, and dry skin occurred less frequently in Black people than in others. This trend was observed consistently throughout the 10 cycles (Figure 2D).

Peripheral sensory neuropathy was found less frequently in Asian people than others, whereas hypertension was observed more frequently in Asian people than others.

### 3.4 Mean grade of toxicities during the 10 cycles

**FIGURE 3:**
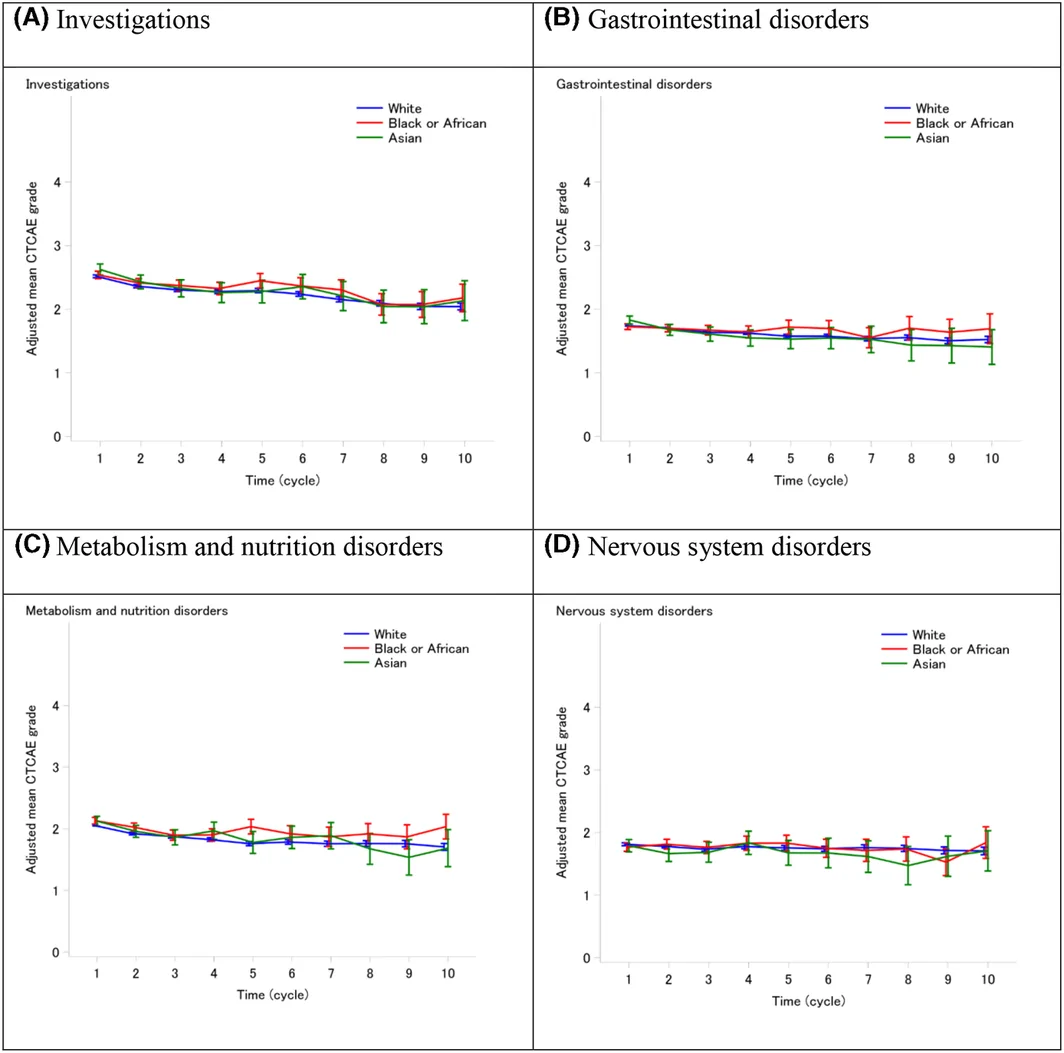
Mean grade of toxicities based on CTCAE during the 10 cycles based on a mixed model for repeated measures. The adjusted mean grades over cycle based on the mixed model for repeated measures (MMRM) that included age (continuous), sex (male vs. female), PS (0–1 vs. ≥2), trial phase (1 vs. 1/2 vs. 2), race, cycle, and interaction of race and cycle. The highest grade toxicity was only counted once at each cycle per patient for each toxicity.

The adjusted mean CTCAE grades in each cycle during 10 cycles by MMRM are shown in Figure 3A–D. Overall, the adjusted mean CTCAE grades in each SOC of toxicities including investigations were noted to gradually decrease over time regardless of race. However, an almost plateaued adjusted mean CTCAE grade over time in gastrointestinal disorders and metabolism and nutrition disorders was shown in Black people. Regarding nervous system disorders, the severity of toxicity of the first cycle in White and Black people tended to remain the same until the 10th cycle.

### 3.5 Time to first grade 2 or higher event

**FIGURE 4:**
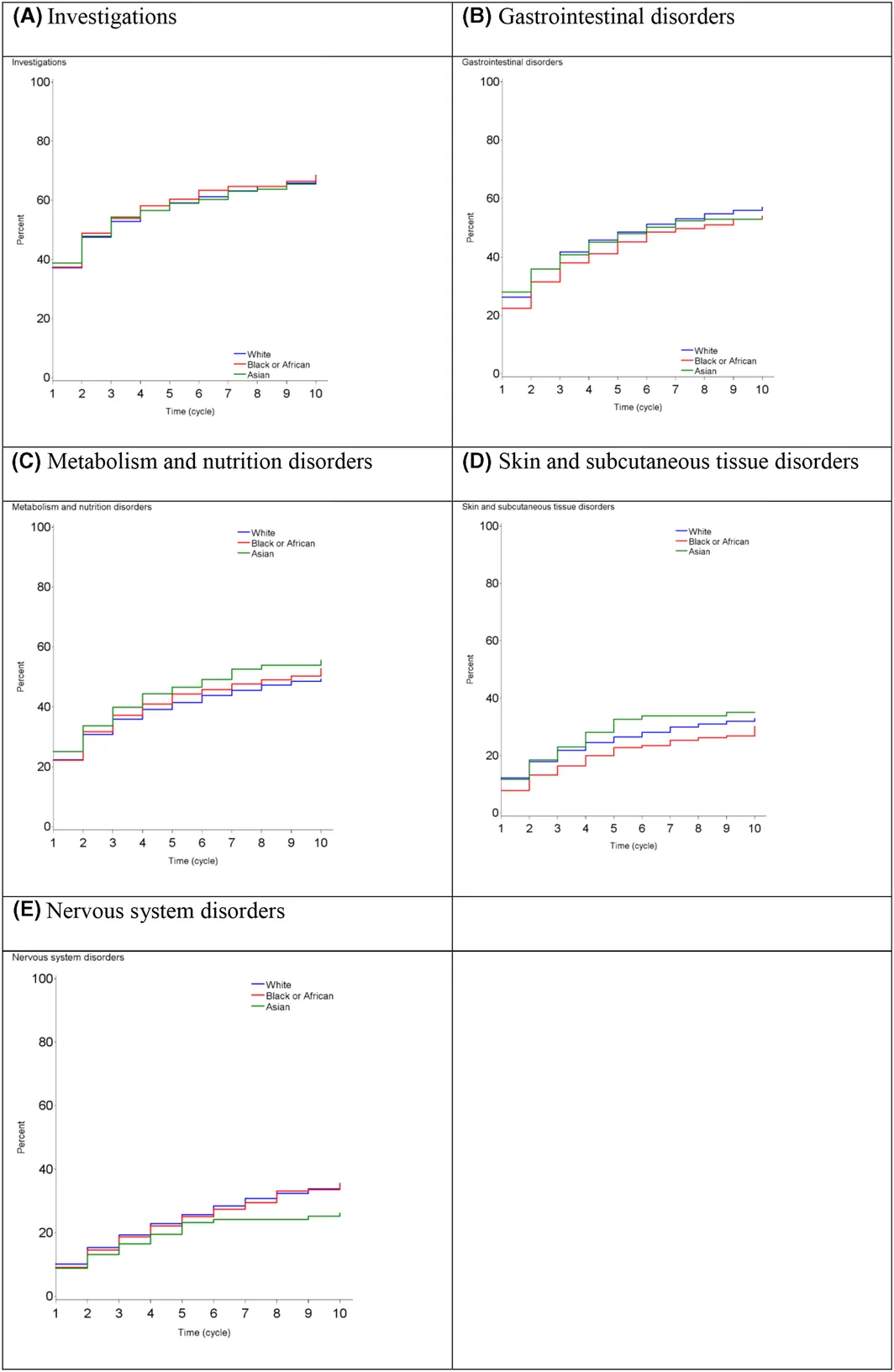
Time to the first grade 2 or higher event. The cumulative proportion of patients who experienced grade 2 toxicity is based on the Kaplan–Meier method. The patients who did not experience the **≥**grade 2 toxicity. during the 10 cycles are censored at the cycle that patients went off‐study.

Figure 4A–E presents the Kaplan–Meier curves of time to the first grade 2 or higher event for each SOC by race. The median time to the first grade 2 or higher event in terms of investigations was 3 cycles irrespective of race. As for gastrointestinal disorders, the median time to the first grade 2 or higher event was 6 cycles (95% confidence interval, not estimable—not estimable) in White people, 8 cycles (95% confidence interval, 6–10) in Black people, and 6 cycles (95% confidence interval, 5—not estimable) in Asian people. The median time to the first grade 2 or higher event in terms of metabolism and nutrition disorders was 7 cycles (95% confidence interval, 5–10) in Asian people and 10 cycles (95% confidence interval, 8—not estimable) in Black people, whereas it was not reached in White people. The median time to the first grade 2 or higher event in terms of skin and subcutaneous tissue disorders was not reached, while the cumulative incidence for Black people tended to be lower compared with White and Asian people. On the other hand, the cumulative incidence for Asian people was clearly low compared with others in terms of nervous system disorders, although the median was not reached.

## 4 DISCUSSION

Using the large database of NCI‐CTEP‐sponsored investigator‐initiated early phase cancer clinical trials, we compared the severity of each treatment cycle, type of AE, mean grade of CTCAE over time, and time to onset of grade 2 or higher AEs across races. Some noteworthy observations were made in this study. The incidence of diarrhea of any grade was found to be 26% and 21% lower in Black people compared to White and Asian people, respectively. The median time to the first occurrence of grade 2 or higher toxicity differed among racial groups, with 6 cycles for White people, 8 cycles for Black people, and 6 cycles for Asian people. Additionally, Asian people exhibited a significantly higher incidence of hyperglycemia of any grade. The strength of this database is that it includes published and unpublished data from both positive and negative outcome trials, reducing the potential for publication bias. The results of this study may serve as foundational data on the frequency, timing, and severity of adverse event occurrence among different racial groups, which could be useful in considering appropriate dose adjustments and treatment modification criteria. To our knowledge, this is one of the largest studies to examine differences in longitudinal toxicity among racial groups using early phase cancer clinical trial data.

The incidence of chemotherapy‐induced diarrhea associated with modulated FU regimens, single‐agent Irinotecan, and the combination of FU plus Irinotecan has been reported elsewhere to be as high as 50%–80% of treated patients, and ≥ 30% of patients may experience grade 3–5 diarrhea.^23^ Racial differences in diarrhea have been reported in several studies of fluoropyrimidines in gastrointestinal cancers.^9^, ^24^ These findings are concordant with our results that among SOC gastrointestinal disorders, diarrhea was about 20% less frequent in Black people than in others. The potential reasons for these differences were variations among races in terms of pharmacokinetics and pharmacodynamics and resulting metabolites; however, the exact causes are not clear.^25^ Furthermore, the frequency of DYPD*2 point mutation and UGT1A1*28 variant was noted to be different among races, but no clear relationship was observed.^26^, ^27^, ^28^, ^29^, ^30^, ^31^, ^32^ Although the rates of observing grade 2 or higher gastrointestinal disorders of SOC adverse events were almost identical among races, Black people tended to incur the toxicities later in the treatment. This is a new finding not previously reported. Overall, further studies with independent data are warranted, to identify the factors associated with the difference among races in terms of diarrhea and other gastrointestinal disorders.

Among metabolism and nutrition disorders, hyperglycemia was significantly higher in Asian people in our study. Although there was a trend, the severity of toxicities was relatively low and reversible. In addition, by longitudinal analyses, Black people tended to have higher severity of hyperglycemia after 8 cycles of therapy. A previous study on prostate cancer found that the frequency of hyperglycemia (in all grades and in grades 3–4) was higher in Black people compared to others (26% vs. 14% in all grades, 10% vs. 4% in grades 3–4).^33^ Fukasawa et al. reported that the incidence of grade 3 hyperglycemia occurring in the abiraterone acetate plus prednisone regimen was higher in the Japanese subpopulation than the overall population (23% vs. 13% in all grades, 11% vs. 5% in grade 3–4) in a randomized phase III trial for patients with metastatic, hormone‐sensitive prostate cancer.^34^, ^35^

It is known that East Asian populations are more likely to develop type 2 diabetes than Western populations, even in the absence of obesity; moreover, type 2 diabetes is a serious disease that increases the risk of various diseases due to chronic hyperglycemia. Recently, molecular biological pathways that are common or different between Japanese and Western populations in the inheritance of type 2 diabetes were identified.^36^ Moreover, following a large‐scale meta‐analysis of genome‐wide association of a 400,000 East Asian population, 61 new genetic regions were found to increase the risk of developing type 2 diabetes.^37^ Such racial differences in genetic information may be associated with the frequency of hyperglycemia during treatment.

One of the strengths of our study is that it provides novel insights on how adverse events differ among populations and an understanding of the potential cumulative effects by using longitudinal graphs. These graphical methods, called the ToxT analysis, were proposed by Thanarajasingam et al. in order to present a more comprehensive visual output of adverse events data instead of just a traditional maximum grade table.^38^, ^39^, ^40^, ^41^ This approach was particularly useful when considering subgroups that may differ in the timing and pattern of adverse events. For example, the timing of grade 2 or higher adverse events for gastrointestinal disorders differed among racial groups, although differences were not apparent if only the worst values were tabulated. It is hoped that publication of the results of ToxT analyses will be commonplace, not only by race, but also by age, sex, and other subgroups that may be affected by toxicity, and that the results will ultimately be used in daily clinical practice.

In recent years, one of the problems in the United States has been the low enrollment of underrepresented populations, such as Black people, in clinical trials.^42^, ^43^, ^44^, ^45^ In our study, about 90% of the subjects in the analysis were White, and only 7% and 3% were Black and Asian people, respectively. This finding was consistent with the previous literature review of phase I oncology trials.^46^ Considering that, in relation to the US cancer incidence, Black people should be enrolled at a prevalence of about 14% and Asian people at about 4%; this study was clearly underrepresented with respect to Black people.^47^ The NIH, FDA, and others have all recognized the importance of this issue^48^, ^49^; accordingly, it is important to enroll underrepresented populations in clinical trials as there are racial differences in clinical responses and toxicity related to therapeutics. According to a systematic review by Unger et al., the participation rate in clinical trials, when offered, was found to be 55%, and this participation rate did not vary significantly among Black, Hispanic, Asian, and White people.^50^ This suggests that individuals, regardless of race, are willing to participate in clinical trials if appropriately invited. Differences in participation rates are also attributed to financial barriers such as medication costs, insurance coverage, and logistical issues related to transportation to healthcare facilities.^51^ It is recognized that addressing the social determinants of health (SDOH) as defined by the World Health Organization is crucial in overcoming these barriers.^52^ The problem is not limited to race; to ensure representation of the target population in terms of age, ethnicity, and other factors, it is necessary to select participating facilities that mirror the actual population distribution and promote decentralized clinical trials that allow participation regardless of residential location.

As a retrospective analysis, this study has several limitations. First, the data used for the study were from early phase clinical trials, which include a wide variety of cancer types. However, because early development trials enroll patients without limiting to specific cancer types, differences in safety by type of cancer are expected to be relatively small compared to efficacy. Second, the limited background information collected means that factors that could influence toxicity, such as pretreatment laboratory values and genetic mutations, were not taken into account in this analysis. We acknowledge that information such as cancer type, medical history, and comorbidities may influence the risk of adverse events. However, the lack of this information leaves the possibility that the adjustment for background factors in assessing the risk of adverse events may not have been adequately performed. In addition, although immune checkpoint inhibitors have dominated cancer therapy development in recent years, these drugs were not included in this present study because the time frame of the study was between 2000 and 2012. The fact that the clinical trials used in this study were conducted in an earlier period may pose challenges in generalizing the results, as the development of immunotherapy and Chimeric Antigen Receptor‐T cell therapy has been advancing rapidly in the current research landscape. Also, reporting bias may exist for subjective toxicity and the heterogeneity of enrollment criteria and treatments in each of the individual studies limits the interpretability of these findings. Furthermore, it can be noted as a limitation that racial classification is very ambiguous and overly broad. For example, in cases where one of the parents of a patient is White and the other is Black, the patient may be considered as a combination of White and Black. However, since the patient generally was classified into only one category, this ambiguity could potentially impact the results of this study.^14^ In addition, it has been pointed out that Asian people are a highly heterogeneous group that can include numerous countries and regions of origin,^53^ and it may not be appropriate to analyze them as a single population. Finally, due to the retrospective nature of these analyses, the observations must be viewed as descriptive and hypothesis generating and should be followed by appropriate prospectively defined investigations. Adverse events reported by patients are influenced by the relationship and cultural background between healthcare providers and patients, which may lead to variations in the frequency and severity of reported events especially for underrepresented populations.

In summary, we identified some notable racial differences in longitudinal toxicities using this large‐scale clinical database. Further study is indicated to clarify the causes of racial differences in toxicities. It is important in the early development phase to interpret and report results in light of the presence of specific racial differences in certain adverse events; accordingly, presentation of more detailed toxicity profiles is warranted.

## AUTHOR CONTRIBUTIONS

**Junki Mizusawa:** Conceptualization (lead); formal analysis (lead); methodology (lead); writing – original draft (lead). **Hiroyuki Sato:** Methodology (equal); writing – review and editing (equal). **Larry V. Rubinstein:** Writing – review and editing (equal). **Takeo Fujiwara:** Project administration (equal); writing – review and editing (equal). **Kan Yonemori:** Project administration (equal); writing – review and editing (equal). **Akihiro Hirakawa:** Conceptualization (equal); formal analysis (equal); methodology (equal); project administration (equal); writing – review and editing (equal).

## FUNDING INFORMATION

This work was partially supported by the Japan Society for the Promotion of Science (Grant No. 20K11703) (“Grant‐in‐aid for scientific research C”).

## CONFLICT OF INTEREST STATEMENT

There are no conflicts of interest to declare related to the submitted work.

## ETHICS STATEMENT

This study was approved by the institutional review board of the National Cancer Center Japan with study number 2015‐143.

## Supporting information


Table S1.

## Data Availability

The research data are available by request to the National Cancer Institute (NCI).
