# Supplementary material for: Racial differences in longitudinal toxicities of anticancer agents in early phase cancer clinical trials
Source: Cancer Med. 2023 Jul 31;12(17):18098–109. doi: 10.1002/cam4.6370 (PMC10524029; doi:10.1002/cam4.6370)
Supplement: Supplementary file 1 — Table S1. [file CAM4-12-18098-s001.docx]

## Supplementary table 1: Drug list

The 68 drugs, comprising 21 CDs and 47 MTAs we analyzed.

| Classification | Drug |
| --- | --- |
| CD | Becatecarin |
| CD | Cisplatin |
| CD | Decitabine |
| CD | Docetaxel |
| CD | Dolastatin |
| CD | Eribulin |
| CD | Fluorouracil |
| CD | Gemcitabine |
| CD | Iododoxorubicin |
| CD | Irinotecan |
| CD | Irofulven |
| CD | Ixabepilone |
| CD | Mitoxantrone |
| CD | Nelarabine |
| CD | Oxaliplatin |
| CD | Paclitaxel |
| CD | Pemetrexed |
| CD | Pyrazoloacridine |
| CD | Pyrrolobenzodiazepine |
| CD | Temozolomide |
| CD | Topotecan |
| MTA | Aflibercept |
| MTA | Alemtuzumab |
| MTA | Alvocidib |
| MTA | Apolizumab |
| MTA | Bevacizumab |
| MTA | Bortezomib |
| MTA | Cabozantinib |
| MTA | Cediranib |
| MTA | Cetuximab |
| MTA | Cixutumumab |
| MTA | Dasatinib |
| MTA | Dihydrochloride |
| MTA | Dinaciclib |
| MTA | Entinostat |
| MTA | Erlotinib |
| MTA | Etracizumab |
| MTA | Everolimus |
| MTA | Fostamatinib |
| MTA | Gefitinib |
| MTA | Imatinib |
| MTA | Ispinesib |
| MTA | Lapatinib |
| MTA | Linsitinib |
| MTA | LMB-2 [Anti-Tac(Fv)-PE-38] |
| MTA | LMB-9 Immunotoxin |
| MTA | Navitoclax |
| MTA | Obatoclax mesylate |
| MTA | Oblimersen |
| MTA | Olaparib |
| MTA | Pazopanib |
| MTA | Perifosine |
| MTA | Rituximab |
| MTA | Saracatinib |
| MTA | Selumetinib |
| MTA | Semaxanib |
| MTA | Sorafenib |
| MTA | Sunitinib |
| MTA | T101 MoAb (anti-human T-lymphocyte antigen 65) |
| MTA | Tandutinib |
| MTA | Temsirolimus |
| MTA | Tipifarnib |
| MTA | Tivantinib |
| MTA | Torebananib |
| MTA | Trastuzumab |
| MTA | Veliparib |
| MTA | Vismodegib |
| MTA | Vorinostat |
